# Phenolic Content and Antioxidant Activity in Raw and Denatured Aqueous Extracts from Sprouts and Wheatgrass of Einkorn and Emmer Obtained under Salinity

**DOI:** 10.3390/molecules22122132

**Published:** 2017-12-02

**Authors:** Beatrice Falcinelli, Paolo Benincasa, Isabella Calzuola, Lilia Gigliarelli, Stanley Lutts, Valeria Marsili

**Affiliations:** 1 Dipartimento di Scienze Agrarie, Alimentari ed Ambientali, Università di Perugia, Perugia 74-06121, Italy; beatricefalcinelli90@gmail.com; 2 Nutraceutical & Phytochemical Products (NPP) S.r.l, Strada Madonna del Giglio, Perugia 15-06132, Italy; isabella.calzuola@npp-srl.com; 3 Dipartimento di Biologia Cellulare e Molecolare, Università di Perugia, Perugia 74-06121, Italy; liliagigliarelli@libero.it (L.G.); valeria.marsili@unipg.it (V.M.); 4 Groupe de Recherche en Physiologie Végétale (GRPV), Earth and Life Institute- Agronomy (ELI-A), Université catholique de Louvain, Croix du Sud 45, boite L7.07.13, Louvain-la-Neuve B-1348, Belgium; stanley.lutts@uclouvain.be

**Keywords:** sprouting, salinity, reducing power, radical scavenging, TBARS

## Abstract

Total phenolic content (TPC), reducing power (RP), superoxide radical scavenging (RS), and thiobarbituric acid reactive substances (TBARS) production inhibition were measured in raw and denatured aqueous extracts from sprouts and wheatgrass of einkorn and emmer obtained at increasing salinity. Grains were incubated and kept at 0, 25, 50, and 100 mM NaCl until either sprout or wheatgrass stage. Additionally, a recovery treatment was included, in which sprouts obtained at 100 mM NaCl were then transferred at 0 mM NaCl until wheatgrass stage. All parameters (TPC, RP, RS, and TBARS production inhibition) increased with sprouting and were highest in wheatgrass. Salinity increased all parameters, but the effect varied with NaCl concentration, genotype, developmental stage, and plant material processing (raw or denatured). Overall, given the delay and limitation of growth at high NaCl concentration, the best compromise appears to be the application of a moderate salinity (25 to 50 mM NaCl). In denatured extracts, TPC, RP, and RS slightly decreased, and TBARS was not affected, which means that antioxidant activity was mainly related to compounds other than enzymes and peptides, and thus it can be assumed to remain after digestion. Thus, supplementing the human diet with einkorn or emmer sprouts and wheatgrass can actually benefit health.

## 1. Introduction

Sprouted grains and wheatgrass of cereals are more and more widespread in the human diet, with a steep increase in western countries. Compared to grains, sprouts and wheatgrass can be consumed raw in salads or used to extract juices without any kind of cooking process, which preserves the integrity of molecules having high nutritional value. Cereals grains are rich in vitamins (E and C), phytochemicals such as polyphenols (phenolic acids, lignans and flavonoids) [1] and carotenoids (lutein, zeaxanthine and β-carotene) [2], and peptides [3]. These low molecular weight substances have several healthy functions, such as antioxidant, anti-arthritic, anti-ulcer, and anticancer activity [3,4]. The scavenging of Reactive Oxygen Species (ROS) has been proven to prevent oxidative damage to DNA in vitro [5].

In cereals, as in other species, the phytochemical content increases during germination and initial seedling growth [3,6]. Benincasa et al. [7] observed a high increase of phenolic compounds in hulled and non-hulled wheat species passing from the sound seed to sprout and then to wheatgrass, with einkorn and emmer showing the highest contents compared to spelt and soft and durum wheat. 

Phenolics and other phytochemicals are secondary metabolites that plants produce to attract pollinators through flower pigmentation, and they also play a major role in defence against biotic or abiotic stresses. For example, salinity causes oxidative stress and excess of ROS [8], and plants react to salt stress by increasing the content of polyphenols and other antioxidants [9]. For this reason, germination under salinity has been proposed as a means to improve the nutritional value of sprouts in broccoli [10], radish [11], rapeseed [12], and buckwheat [13].

In all these works, the polyphenol content was measured on alcoholic or hydro-alcoholic extracts for obtaining the highest amount of extractable molecules. However cereal sprouts and wheatgrass are consumed as salads or juices [7], i.e., they include their own constitutive water and no alcohol. Thus water extracts are nutritionally more meaningful than alcoholic or hydro-alcoholic ones. Moreover, water gives numerous advantages for certification and safety being an environmentally safe and non-toxic alternative, which allows to extract all hydrophilic antioxidants [14]. 

Finally, in most works on sprouts, the antioxidant activity is measured on crude extracts, but this does not necessarily correspond to that available after digestion because the passage through the stomach and intestine may alter antioxidant components. On one hand, the use of a crude extract as an additive needs to be considered so that the sensory properties of the food product are not adversely affected [14]. On the other hand, heating sprouts and wheatgrass extracts up to 80 °C may denature enzymatic proteins having antioxidant activity, so that the antioxidant activity of denatured extracts would concern other antioxidants that likely reach the human intestine [15].

Therefore, the objectives of this research were to study the polyphenol content and antioxidant activity of raw and denatured aqueous extracts from sprouts and wheatgrass of einkorn and emmer obtained at increasing levels of salinity in the germination substrate. This in order to (i) support sprouting under salinity as a means to increase the nutritional value of sprouts; and (ii) individuate the nature of the compounds (i.e., low or high molecular weight; thermally stable or not) implicated in the antioxidant activity of sprouts. 

## 2. Results

### 2.1. Germination and Growth Performance

The percent germination was not substantially affected by NaCl concentration in both genotypes, whereas T50 increased in S50 and S100 especially in *Triticum dicoccum* cv Zefiro (TDiZ), where two days delay was recorded for S100 as compared to S0 (Table 1).

Seedling growth was as slower as higher was the salinity, but while most individuals of *Triticum monococcum* cv Monlis (TMoM) S100 reached the wheatgrass stage, only 11% succeeded in TDiZ S100; the rest of them remained small and weak, and actually stopped growing (Table 1).

### 2.2. Total Phenolic Content

**Figure 1:**
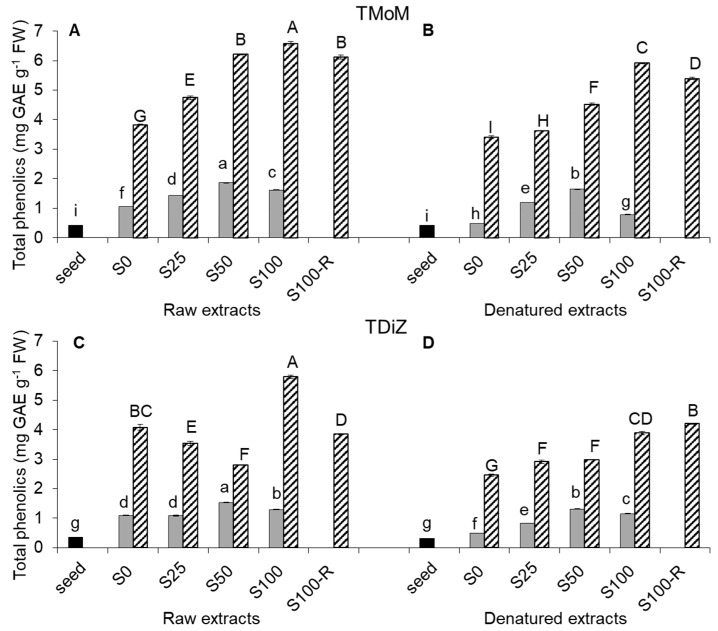
Total phenolic content (TPC; mg gallic acid equivalents g^−1^ fresh weight) in raw and denatured extracts from seeds (black), sprouts (grey), and wheatgrass (zebra-striped) of *Triticum monococcum* cv Monlis (TMoM) and *Triticum dicoccum* cv Zefiro (TDiZ) sprouted and grown with salt (NaCl) concentration 0, 25, 50, 100 mM (S0, S25, S50, S100) or sprouted with 100 mM and then grown with distilled water as recovery treatment (S100-R). Values are normalized for a dry matter content of 150 mg g^−1^. Average values of triplicate determinations for *n* = 2 independent replicates ± standard error are depicted. Different letters indicate statistically significant differences at *p* < 0.05 (Fisher’s LSD).

The total phenolic content (TPC) of raw extracts increased with the developmental stage in both TMoM (Figure 1A) and TDiZ (Figure 1C). 

On average over NaCl treatments, TPC of sprouts was over three-fold compared to seeds, and that of wheatgrass was over three-fold compared to sprouts. Salinity increased TPC in both sprouts and wheatgrass. The maximum TPC in both species was recorded for S50 in sprouts (+71% in TMoM and +39% in TDiZ, as compared to S0) and S100 in wheatgrass (+72% in TMoM and +42% in TDiZ). However, in TDiZ, NaCl concentrations lower than 100 mM decreased TPC compared to S0. In both species, S100-R showed TPC lower than S100, but the value was much lower for TDiZ. 

In denatured extracts (Figure 1B,D), the TPC of any treatment was generally lower than in the raw extract of the corresponding treatment. However, relative differences among treatments remained roughly similar as in raw extracts. Only TDiZ wheatgrass for S50 and S100-R showed a non-significant increase of TPC after denaturation.

### 2.3. Reducing Power

**Figure 2:**
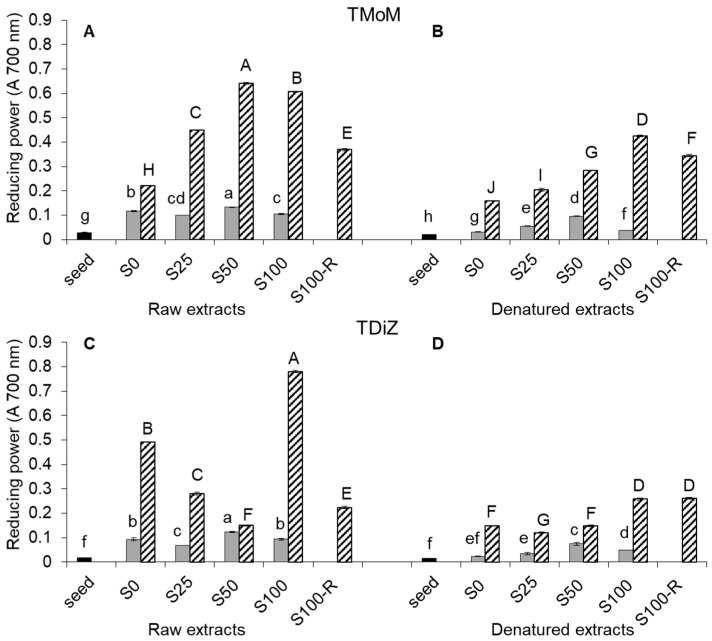
Total reducing power (RP) in raw and denatured extracts from seeds (black), sprouts (grey), and wheatgrass (zebra-striped) of *Triticum monococcum* cv Monlis (TMoM) and *Triticum dicoccum* cv Zefiro (TDiZ) sprouted and grown with salt (NaCl) concentration 0, 25, 50, 100 mM (S0, S25, S50, S100) or sprouted with 100 mM and then grown with distilled water as recovery treatment (S100-R). All values are referred to a concentration of 143 mg/mL. Average values of triplicate determinations for *n* = 2 independent replicates ± standard error are depicted. Different letters indicate statistically significant differences at *p* = 0.05 (Fisher’s LSD).

The reducing power (RP) of raw extracts increased with the developmental stage in both TMoM (Figure 2A) and TDiZ (Figure 2C). 

On average over the NaCl concentrations, RP of sprouts was about four and five times higher than in seeds for TMoM and TDiZ, respectively, and RP of wheatgrass was four times higher than in sprouts for both species.

Concerning salinity, a slight increase of RP in sprouts of both species was observed only for S50, while the highest RP in wheatgrass was recorded for S50 in TMoM and S100 in TDiZ. As for TPC, RP in TDiZ wheatgrass decreased at NaCl concentrations lower than 100 mM compared to the control. In both species, S100-R showed RP lower than S100. 

In denatured extracts (Figure 2B,D), RP values were generally lower than in raw extracts for any correspondent NaCl treatment, except for a moderate increase in S100-R wheatgrass of TDiZ. The trends across salt treatments were similar to those recorded for TPC.

### 2.4. Radical Scavenging

**Figure 3:**
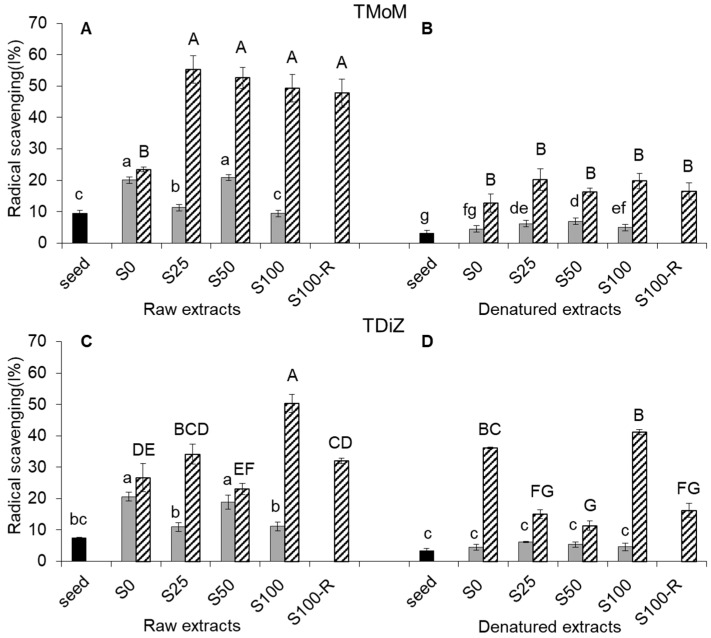
Radical scavenging (RS) activity (%) in raw and denatured extracts from seeds (black), sprouts (grey), and wheatgrass (zebra-striped) of *Triticum monococcum* cv Monlis (TMoM) and *Triticum dicoccum* cv Zefiro (TDiZ) sprouted and grown with salt (NaCl) concentration 0, 25, 50, 100 mM (S0, S25, S50, S100) or sprouted with 100 mM and then grown with distilled water as recovery treatment (S100-R). All values are referred to a concentration of 143 mg/mL. Average values of triplicate determinations for *n* = 2 independent replicates ± standard error are depicted. Different letters indicate statistically significant differences at *p* = 0.05 (Fisher’s LSD).

As for TPC and RP, the radical scavenging (RS) of raw extracts increased passing from sound seeds to sprouts to wheatgrass for both species (Figure 3A,C).

On average over salt treatments, RS of sprouts in TMoM was +61% than in sound seeds, and RS of wheatgrass was almost triple than in sprouts. In TDiZ, RS doubled passing from seeds to sprouts and doubled from sprouts to wheatgrass. Salinity had a dual effect depending on the developmental stage and NaCl treatment. In sprouts of both species, RS fluctuated from high values of S0 and S50 and low values of S25 and S100. In wheatgrass of TMoM, RS was lowest in the unsalted control and increased markedly in all the other treatments, whereas in wheatgrass of TDiZ, only the increase recorded for S100 was significant and a marked drop was recorded in S100-R.

In denatured extracts, RS values were generally lower than in raw extracts for any correspondent NaCl treatment (Figure 3B,D), except for the S0 wheatgrass of TDiZ. 

### 2.5. TBARS Production Inhibition

**Figure 4:**
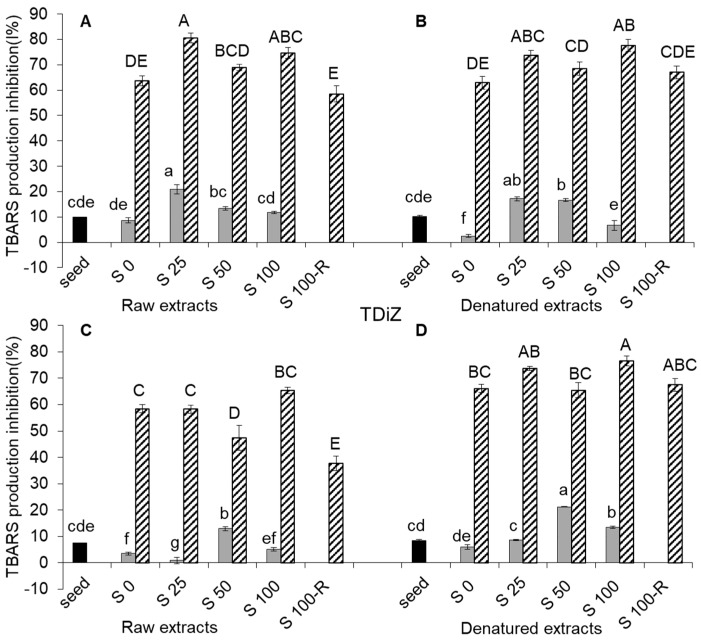
Thiobarbituric acid reactive substances (TBARS) production (lipid peroxidation) inhibition (%) in raw and denatured extracts from seeds (black), sprouts (grey), and wheatgrass (zebra-striped) of *Triticum monococcum* cv Monlis (TMoM) and *Triticum dicoccum* cv Zefiro (TDiZ) sprouted and grown with salt (NaCl) concentration 0, 25, 50, 100 mM (S0, S25, S50, S100) or sprouted with 100 mM and then grown with distilled water as recovery treatment (S100-R). All values are referred to a concentration of 143 mg/mL. Average values of triplicate determinations for *n* = 2 independent replicates ± standard error are depicted. Different letters indicate statistically significant differences at *p* = 0.05 (Fisher’s LSD).

Thiobarbituric acid reactive substances (TBARS) production inhibition in raw extracts for both species was slightly affected passing from sound seeds to sprouts, whereas it greatly increased from sprouts to wheatgrass (over five-fold in TMoM and over eight-fold in TDiZ, on average over salt treatments) (Figure 4A,C). 

The effect of salinity did not follow a clear trend. In sprouts, TBARS production inhibition was highest for S25 in TMoM and S50 in TDiZ (+216% and +369%, respectively, compared to S0), but was almost null for S25 in TDiZ. In wheatgrass, TBARS production inhibition showed little variations across salt treatments. In both species, S100-R showed a significant decrease compared to S100. 

TBARS production inhibition was not significantly reduced by denaturation in TMoM (Figure 4B), except in the case of sprouts for S0, and was generally increased in sprouts and wheatgrass of TDiZ, significantly for S25, S50 and S100-R (Figure 4D).

## 3. Discussion

In both species, increasing salinity slowed germination and growth and, in case of TDiZ wheatgrass, 100 mM NaCl severely compromised biomass yield (data not shown) because most individuals stopped growing (Table 1). This suggests that 100 mM NaCl is too high for sprout production purpose. Data on the T50 and time to achieve wheatgrass stage indicate that TMoM was more salt tolerant than TDiZ. The uneven response of TDiZ individuals in S100 is not easy to explain because this cultivar is a pure line, expected to be genetically uniform from a theoretical point of view. However, a limited genetic variability can still occur for these less bred species, especially for genes that could be induced in response to stress. In fact, homogeneity was selected by breeders mostly for optimal growth and high yield under non-stress conditions, but variability may still remain for genes that play a key role in response to high stress intensities. Moreover, the response may vary with the seed lot, and finally, any lot is known to consist of percentiles with different base osmotic potential [16], which likely affects also initial growth. The individuals of TDiZ S100 that reached the wheatgrass stage represent a sort of “champions of the seed lot” which likely owed their salt tolerance to molecular and biochemical issues.

The increase of TPC observed in raw extracts (Figure 1A,C) passing from the seed to sprouts to wheatgrass confirms evidences obtained in the same genotypes [7], as well as in wheat [17]. However, the values obtained in our study are much greater than those reported by Benincasa et al. [7], but they measured TPC on methanol extracts. 

The increase in TPC complies with the need of seedlings to prevent damages during early growth [18]. In fact, phenolic compounds are produced by plants through the phenylpropanoid pathway, as a reaction against environmental stresses or elicitors [19]. This explains also the increase in TPC we observed in salted treatments, since salinity stimulates phenylalanine ammonia lyase (PAL), the key synthesis enzyme of phenolic compounds involved in phenylpropanoid pathway [20], and thus leads to the polyphenol accumulation [12,13]. By contrast, Yuan et al. [11] and Guo et al. [10] found a decreasing phenolic content at increasing NaCl concentrations in radish and broccoli sprouts. Differences may depend on the species, the growth stage, and the extraction solvent (alcoholic solutions vs. water). The positive TPC vs. NaCl dose response trend observed at the sprout stage in both genotypes is in line with that recorded in buckwheat sprouts [13]. The different trend observed in the two genotypes at the wheatgrass stage may arise from a different salt sensitivity revealed by the prolonged exposure to salinity. Our results in einkorn wheatgrass were similar to those obtained in buckwheat after seven days from germination [13] and the high TPC value recorded in the recovery treatment (100-R) suggests that this genotype keeps ready to face stressing conditions. The decreasing trend recorded in TPC of emmer wheatgrass from S0 to S50 could be due to its higher sensitivity to salt stress, in agreement with its slower growth. On the other hand, the great increase of TPC observed in emmer wheatgrass in S100 can be explained with the presence of the above said “champions of the seed lot”, i.e., the few individuals able to reach the wheatgrass stage. In fact, the individuals unable to survive or grow until wheatgrass stage in S100, i.e., those with a likely low TPC content, were not included in the sample. The low TPC recorded for 100-R would support this hypothesis. In these treatment, in fact, almost all individuals reached the sprout stage and, when transferred to the unsalted substrate, could then be harvested as wheatgrass. These results confirm evidences from other studies: salinity could improve phenolic compounds synthesis but it is closely related to genotype salt sensitivity and to salt concentration [21]. 

The decrease of TPC after denaturation is in line with literature (Figure 1B,D). However it is worth to pinpoint that the drop of TPC reported by some authors [22,23] could be due to the loss of polyphenols in cooking water, while we prevented this leaching because extracts were contained in falcons during the denaturation in the water bath (see Section 4.2). As said above, the Folin–Ciocalteu reagent is not specific just to polyphenols, thus the decrease of TPC we observed in our extracts could be due to the phenolic breakdown during cooking [24], but also to the degradation of other molecules that reacted with the Folin–Ciocalteu reagent. The effects of thermal processing depends on polyphenols concentration, chemical structure, oxidation, localization in the cell, interaction with other food components, and type of thermal processing applied [25]. In fact, Turkmen et al. [26] observed losses and increases of polyphenols depending on plant species and cooking method. 

The increase in RP of raw extracts observed with sprouting until the wheatgrass stage confirms evidences in wheat [3], green and horse gram [27], and sesame [28] (Figure 2A,C). The effect of salinity was little in sprouts as compared to that observed for TPC, greater in wheatgrass, where, the trend for each of the two genotypes was similar to that observed for TPC, except for a more marked drop in 100-R. This suggests that the reducing power in wheatgrass was partly due to the presence of hydrophilic antioxidants other than polyphenols, highly produced passing from sprouts to wheatgrass and in the presence of salt. The greater drop of RP (compared to that of TPC) observed in wheatgrass at 100-R would support this hypothesis. Calzuola et al. [3] found that aqueous extracts of wheat sprouts showed a reducing activity around 10 times higher than that exerted by ethanolic extracts, probably due to the presence in aqueous extracts of some enzymes involved in the ferrycyanide reduction. The general decrease of RP observed in denatured extracts (Figure 2B,D) indicates that part of RP was due to compounds that are not thermally stable. 

As far as RS is concerned, the lack of effect of salinity in sprouts (compared to the important effect on TPC) suggests that, besides polyphenols, other compounds assume key functions in the management of oxidative stress and that salinity allows to increase these compounds as well (Figure 3A,C). On the contrary, for wheatgrass, even a low salinity level was enough to promote the maximum RS in the salt tolerant genotype (TMoM) and the effect lasted after the removal of salinity (100-R). Conversely, no significant increase of radical scavenging was recorded for the salt-sensitive genotype (TDiZ) except in S100, but again this can be explained with the contribution of the few salt tolerant individuals grown until wheatgrass stage, as discussed previously for TPC. Superoxide dismutase (SOD), the specific enzyme that catalyzes the disproportion of superoxide, has been reported to increase during germination [29] and in the presence of salt stress [20], but the effect would change with salt sensitivity of genotypes [30,31]. The decrease observed after denaturation (Figure 3B,D) suggests that part of RS activity was due to thermally unstable compounds, like peptides [3]. On the other hand, despite this decrease, RS, as well as RP, kept considerable after denaturation, suggesting that some antioxidants (likely the phenolic compounds) are thermally stable compounds and can remain available after digestion.

Hydroxyl radical is considered as the most reactive oxidant in cells causing lipid peroxidation and DNA damage [32] and in our study the % inhibition of TBARS production represents the capability of extracts to scavenge OH^–^ [33]. The effect of germination and salinity on lipid peroxidation is well known, but not for einkorn and emmer sprouts and wheatgrass. Our results show that TBARS production inhibition in sprouts of these species was very low and not substantially different from that of seeds whereas it increased much in wheatgrass (Figure 4A,C). The tocols and flavonoids found in einkorn and emmer [2], which are able to scavenge OH^–^, could be responsible for this result. In general, denaturation did not reduce TBARS production inhibition of sprouts and wheatgrass in einkorn (Figure 4B) or even increased it in emmer (Figure 4D). This can be explained only by assuming that TBARS production inhibition arises from thermally stable hydrophilic compounds produced during initial seedling growth and these compounds would be different from those involved in RP and RS. Actually, Jimenèz-Monreal et al. [34] report that the antioxidant activity of some vegetables after cooking may increase due to several possible reasons: the release of antioxidant components after the thermal destruction of cell walls and subcellular compartments; the production of stronger radical-scavenging antioxidants by thermal chemical reaction; the suppression of the oxidation capacity of antioxidants by thermal inactivation of oxidative enzymes; the production of new non-nutrient antioxidants or the formation of novel compounds such as Maillard reaction products having antioxidant activity. Further studies are needed to individuate these compounds, define the biochemical mechanisms that regulate their production and the effect of heat treatment on their activity. 

## 4. Materials and Methods

### 4.1. Plant Material and Experimental Design

Grains of einkorn (*Triticum monococcum* L. ssp. *monococcum* cv. Monlis, TMoM) and emmer [*Triticum turgidum* L. spp. *dicoccum* (Schrank ex Shubler) Thell., cv. Zefiro, TDiZ] harvested from Central Italy crops were incubated in plastic trays containing solutions with 0, 25, 50, and 100 mM NaCl (treatments S0, S25, S50, S100) according to a completely randomized block design with four replicates (trays). Parallel germination tests were performed on Petri dishes (two replicates of 50 grains each) to determine percent germination and time to reach 50% of germinated seeds. Once sprouts were obtained, part of them were harvested and part were kept on growing until wheatgrass stage. Additionally, part of the sprouts from S100 were transferred to 0 NaCl until wheatgrass stage as recovery treatments (S100-R). In order to guarantee constant water availability and prevent anoxia, grains were positioned on filter paper laid over glass balls immersed in the solution contained into the trays. The trays were weighed individually at the beginning of the experiment and distilled water was added daily to restore initial weight and keep NaCl concentration of each treatment constant, in the assumption that changes in plant weight during sprouting were risible as compared to those due to water evaporation [12]. The trays were placed in a growth chamber at 18 °C in dark and, from the third day at a light/dark regime of 10–14 h, with light intensity of 200 μmol photons m^−2^ s^−1^ [7]. In both species, sprouts from the unsalted control (S0) were collected at 5 days after sowing (DAS), with an individual average fresh weight (including roots and the grain residue) of 0.28 g for TMoM and 0.31 g for TDiZ, and shoot length of 53 mm for TMoM and 56 mm for TDiZ. Wheatgrass from the unsalted control was collected at 8 DAS, with an average shoot fresh weight (only the shoot portion) of 0.15 g for both species and shoot length of 116 mm for TMoM and 124 mm for TDiZ. Sprouts and wheatgrass of other treatments were collected when the growth stages corresponded approximately to those of S0: i.e., 5 and 8 DAS for S25; 6 and 9 DAS for S50 in TMoM, and 6 and 11 DAS for S50 in TDiZ; 7 and 10 DAS for S100 in TMoM, and 8 and 13 DAS for S100 in TDiZ; and 9 DAS for S100-R in both genotypes. In TDiZ S100, only few individuals reached the wheatgrass stage at 13 DAS, but the experiment was stopped anyway and only those individuals were harvested, because the remaining seedlings were clearly suffering and had stopped growing. Replicates of each treatment were then re-grouped two by two for the chemical analysis, performed in triplicate. Only individuals that reached the wheatgrass stage were collected for analysis. Samples were stored at −20 °C until extraction.

### 4.2. Sample Preparation

Raw seeds, sprouts, and wheatgrass were homogenized five times with distilled water using a mixer, alternating 30 s of homogenization and 30 s pause to prevent the material from heating. The extract was centrifuged at 7000× *g* for 30 min at 4 °C. The supernatant was stored at −20 °C for analysis. 

To obtain denatured extracts, aliquots of extracts were kept in falcon and immersed in a water bath at 80 °C for 30 min [35]. The precipitated pellet was removed trough centrifugation and the supernatant was stored at −20 °C for analysis.

### 4.3. Chemical Reagents

All reagents were of pure analytical grade. Potassium ferricyanide, thiobarbituric acid (TBA), Folin–Ciocalteu reagent, hypoxanthine, xanthine-oxidase from bovine milk, and nitrotetrazolium blue chloride (NBT) were purchased from Sigma Chemical Co., St. Louis, MO, USA.

### 4.4. Total Phenolic Content

The total phenolic content was measured according to Singleton and Rossi [36] with phosphomolybdic–phosphotungstic acid reagent (Folin–Ciocalteu reagent). The extract (47 μL), or distilled water for the blank, was mixed with 50 μL of Folin–Ciocalteu reagent, 100 μL of Na_2_CO_3_ 200 mg g^−1^ and 803 μL of distilled water. Each sample had its own control, in which Folin–Ciocalteu reagent was replaced with the same quantity of distilled water. The mixture was incubated at room temperature for 30 min and the absorbance was measured at 700 nm. The values were computed by utilizing the dose-response obtained with gallic acid as standard. 

### 4.5. Reducing Power

The total reducing power was measured using potassium ferricyanide as reagent, according to Yen and Chen [37]. The extract was mixed with an equal volume of 0.2 M phosphate buffer, pH 6.6, and 10 mg g^−1^ potassium ferricyanide and incubated at 50 °C for 20 min. An equal volume of 10 mg g^−1^ trichloroacetic acid was added to the mixture, which was then centrifuged at 6000× *g* for 10 min. A portion of the upper layer was mixed with distilled water and 1 mg g^−1^ FeCl_3_ at a ratio of 1:1:2. The absorbance was measured at 700 nm. Increased absorbance of reaction mixture indicated increased reducing power.

### 4.6. Radical (Superoxide) Scavenging

Measurement of superoxide radical scavenging activity was achieved according to Kirby and Schmidt [38]. The reaction mixture was prepared with 25 μL of Na_2_EDTA 15 mM in buffer (50 mM KH_2_PO_4_/KOH, pH 7.4), 62 μL of 0.6 mM NBT in buffer, 37 μL of 3 mM hypoxanthine in 50 mM KOH, 6.25 μL of extract for samples, or distilled water for blanks, and 181 μL of buffer. Two 150-μL samples of reacting mixture were placed in two wells of 96-well microplates (Falcon). 25 μL of distilled water were added to one well, while 25 μL of xanthine oxidase solution (1 unit in 10 mL of buffer) were added to the other well, causing the enzymatic reaction beginning. Then the reaction mixture was immediately incubated at 25 °C, and the absorbance at 550 nm was determined every 1 min in the first 5 min and then every 5 min up to 60 min using a plate reader (Labsystems, MultisKan, MS, USA). The capability to scavenge oxygen superoxide was calculated as inhibition % of oxygen superoxide according to the following equation:

I% = 100 − (A/A_0_ × 100) (1)

where A is the absorbance of the sample and A_0_ is the absorbance of control.

### 4.7. TBARS Production (Lipid Peroxidation) Inhibition

A standardized solution of Fe–EDTA was prepared freshly by mixing equal volumes of EDTA (ethylenediamine tetraacetic acid) 2 mM in sodium phosphate buffer (100 mM, pH 7.4) and Fe(NH_4_)_2_SO_4_ 2 mM in distilled water, according to Koracevic et al. [33]. The solution was incubated in the dark at room temperature for at least one hour to obtain the complex Fe–EDTA. Each sample had its control and treated sample. Samples were prepared with 6.25 μL of extract, or distilled water for blank, 494 μL of sodium phosphate buffer (100 mM, pH 7.4), and 500 μL sodium benzoate (10 mM). Then 1000 μL of acetic acid 200 mg g^−1^ were added only to control samples to stop the reaction, and 200 μL of Fe-EDTA solution and 200 μL of H_2_O_2_ were added to all samples, which were incubated for 60 min at 37 °C. Then, 1000 μL of acetic acid 200 mg g^−1^ were added only to the treated sample, and 1000 μL of thiobarbituric acid (TBA) 10 mM were added to all samples, which were then incubated at 100 °C in a boiling bath for 10 min and cooled in an ice bath. The absorbance was measured at 532 nm. The capability to inhibit TBARS production was calculated as inhibition % of TBARS production, with the Equation (1).

### 4.8. Statistical Analysis

Since plant material water content increased with the developmental stage and decreased with salinity, all data were normalized for a dry matter content of 130 mg g^−1^ (i.e., the average d.m. concentration in the wheatgrass of S0), in order to allow comparisons between stages (i.e., sound grains, sprouts, and wheatgrass) and NaCl treatments. Average values of triplicate determinations for *n* = 2 independent replicates ± standard error are depicted. Means were compared by using the Fisher’s Least Significant Difference (LSD) at *p* = 0.05. 

## 5. Conclusions

Results demonstrate that aqueous extracts from sprouts and wheatgrass of einkorn cv. Monlis and emmer cv Zefiro were rich in polyphenols and other antioxidants. This provides these foods with high levels of reducing power, radical scavenging, and TBARS production inhibition. Salinity increased polyphenols and antioxidant activity in sprouts and wheatgrass of einkorn cv Monlis, and in sprouts of emmer cv Zefiro, but not in its wheatgrass, which suffered from salt stress. In both genotypes, the effect varied with the salinity level and stage of development. Overall, considering that a high NaCl concentration (i.e., 100 mM NaCl) may delay germination and delay or limit growth, we can conclude that a moderate salinity (25 to 50 mM NaCl) may represent the best compromise to maximize polyphenol and antioxidant yield in salt tolerant genotypes. A relevant antioxidant activity remained after denaturation, which means it was related to compounds other than enzymes and peptides, and thus it can be assumed to remain available after digestion. This supports our deduction that supplementing human diet with einkorn or emmer sprouts and wheatgrass can actually benefit health. 

## Figures and Tables

**Table 1:** Germination percentage (G), time to obtain 50% germination (T50), and percentage of individuals that reached the wheatgrass stage in *Triticum monococcum* cv Monlis (TMoM) and *Triticum dicoccum* cv Zefiro (TDiZ) sprouted and grown with 0, 25, 50, 100 mM of salt (S0, S25, S50, S100) or sprouted with 100 mM and then grown with distilled water as recovery treatment (S100-R). Standard errors in brackets.

Genotype	Treatment (mM)	G (%)	T50 (d)	Wheatgrass Individuals (%)
TMoM	S0	82 (1.5)	3.4 (0.1)	96 (1.3)
	S25	84 (0.3)	3.5 (0.2)	99 (1.2)
	S50	85 (1.3)	3.8 (0.1)	96 (1.1)
	S100	82 (0.7)	4.1 (0.2)	94 (1.2)
	S100-R	-	-	97 (1.0)
TDiZ	S0	79 (3.3)	3.6 (0.0)	99 (1.3)
	S25	81 (1.1)	3.5 (0.3)	92 (0.1)
	S50	85 (2.7)	4.2 (0.1)	77 (8.4)
	S100	76 (4.3)	5.5 (0.6)	11 (1.0)
	S100-R	-	-	90 (2.5)

## References

[1] Dykes L., Rooney L.W. (2007). Phenolic compounds in cereal grains and their health benefits. Cereal Food World.

[2] Lachman L., Hejtmankova K., Kotìkovà Z. (2013). Tocols and carotenoids of einkorn, emmer and spring wheat varieties: Selection for breeding and production. J. Cereal Sci..

[3] Calzuola I., Marsili V., Gianfranceschi G.L. (2004). Synthesis of antioxidants in wheat sprouts. J. Agric. Food Chem..

[4] Singh N., Verma P., Pandey B.R. (2012). Therapeutic potential of organic *Triticum aestivum* Linn. (Wheat Grass) in prevention and treatment of chronic disease: An overview. Int. J. Pharm. Sci. Drug Res..

[5] Falcioni G., Fedeli D., Tiano L., Calzuola I., Macinelli L., Marsili V., Gianfranceschi G. (2002). Antioxidant activity of wheat sprouts extract in vitro: Inhibition of DNA oxidative damage. J. Food Sci..

[6] Donkor O.N., Stojanovska L., Ginn P., Ashton J., Vasiljevic T. (2012). Germinated grains—Sources of bioactive compounds. Food Chem..

[7] Benincasa P., Galieni A., Manetta A.C., Pace R., Guiducci M., Pisante M., Stagnari F. (2015). Phenolic compounds in grains, sprouts and wheatgrass of hulled and non-hulled wheat species. J. Sci. Food Agric..

[8] Turkan I., Demiral T. (2009). Recent developments in understanding salinity tolerance. Environ. Exp. Bot..

[9] Waśkiewicz A., Muzolf-Panek M., Goliński P. (2013). Phenolic content changes in plants under salt stress. Ecophysiology and Responses of Plants under Salt Stress.

[10] Guo L., Yang R., Wang Z., Guo Q., Gu Z. (2014). Effect of NaCl stress on health-promoting compounds and antioxidant activity in the sprouts of three broccoli cultivars. Int. J. Food Sci. Nutr..

[11] Yuan G., Wang X., Guo R., Wang Q. (2010). Effect of salt stress on phenolic compounds, glucosinolates, myrosinase and antioxidant activity in radish sprouts. Food Chem..

[12] Falcinelli B., Sileoni V., Marconi O., Perretti G., Quinet M., Lutts S., Benincasa P. (2017). Germination under moderate salinity increases phenolic content and antioxidant activity in rapeseed (*Brassica napus* var *oleifera* Del.) sprouts. Molecules.

[13] Lim J.H., Park K.J., Kim B.K., Jeong J.W., Kim H.J. (2012). Effect of salinity stress on phenolic compounds and carotenoids in buckwheat (*Fagopyrum esculentum* M.) sprout. Food Chem..

[14] Wong S.P., Leong L.P., Koh J.H.W. (2006). Antioxidant activities of aqueous extracts of selected plants. Food Chem..

[15] Hęś M., Dziedzic K., Górecka D., Drożdżyńska A., Gujska E. (2014). Effect of boiling in water of barley and buckwheat groats on the antioxidant properties and dietary fiber composition. Plant Food Hum. Nutr..

[16] Bradford K.J., Kigel J., Galili G. (1995). Water relations in seed germination. Seed Development and Germination.

[17] Alvarez-Jubete L., Wijngaard H., Arendt E.K., Gallangher E. (2010). Polyphenol composition and in vitro antioxidant activity of amaranth, quinoa buckwheat and wheat as affected by sprouting and baking. Food Chem..

[18] Cevallos-Casals B.A., Cisneros-Zevallos L. (2010). Impact of germination on phenolic content and antioxidant activity of 13 edible seed species. Food Chem..

[19] Kim H.J., Chen F., Wang X., Choi J.H. (2006). Effect of methyl jasmonate on phenolics, isothiocyanate, and metabolic enzymes in radish. J. Agric. Food Chem..

[20] Gao S., Ouyang C., Wang S., Xu Y., Tang L., Chen F. (2008). Effects of salt stress on growth, antioxidant enzyme and phenylalanine ammonia-lyase activities in *Jatropha curcas* L. seedlings. Plant Soil Environ..

[21] Kim H.J., Fonseca J.M., Choi J.H., Kubota C., Kwon D.Y. (2008). Salt in irrigation water affects the nutritional and visual properties of romaine lettuce (*Lactuca sativa* L.). J. Agric. Food Chem..

[22] Ismail A., Marjan M.Z., Foong C.W. (2004). Total antioxidant activity and phenolic content in selected vegetables. Food Chem..

[23] Zhang D., Hamauzu Y. (2004). Phenolics, ascorbic acid, carotenoids and antioxidant activity of broccoli and their changes during conventional and microwave cooking. Food Chem..

[24] Crozier A., Lean M.E.J., McDonald M., Black C. (1997). Quantitative analysis of the flavonoid content of commercial tomatoes, onions, lettuce, and celery. J. Agric. Food Chem..

[25] Van Boekel M., Fogliano V., Pellegrini N., Stanton C., Sholz G., Lalijie S., Somoza V., Knorr D., Jasti P.-R., Eisenbrand G. (2010). A review on the beneficial aspects of food processing. Mol. Nutr. Food Res..

[26] Turkmen N., Sari F., Velioglu S. (2005). The effect of cooking methods on total phenolics and antioxidant activity of selected green vegetables. Food Chem..

[27] Ramesh C.K., Rehman A., Prabhakar B.T., Vijay Avin B.R., Aditya Rao S.J. (2011). Antioxidant potentials in sprouts vs. seeds of *Vigna radiata* and *Macrotyloma uniflorum*. J. Appl. Pharm. Sci..

[28] Liu B., Guo X., Zhu K., Liu Y. (2011). Nutritional evaluation and antioxidant activity of sesame sprouts. Food Chem..

[29] Bogdanovic J., Radotic K., Mitrovic A. (2008). Changes in activities of antioxidant enzymes during *Chenopodium murale* seed germination. Biol. Plant..

[30] Esfandiari E., Shekari F., Shekari F., Esfandiari M. (2007). The effect of salt stress on antioxidant enzymes’ activity and lipid peroxidation on the wheat seedling. Not. Bot. Horti Agrobot..

[31] Sairam R.K., Srivastava G.C., Agarwal S., Meena R.C. (2005). Differences in antioxidant activity in response to salinity stress in tolerant and susceptible wheat genotypes. Biol. Plant..

[32] Arora A., Sairam R.K., Srivastava G.C. (2002). Oxidative stress and antioxidative system in plants. Curr. Sci. India.

[33] Koracevic D., Djordjevic V., Andrejevic S., Cosic V. (2001). Method for the measurement of antioxidant activity in human fluids. J. Clin. Pathol..

[34] Jimenèz-Monreal A.M., Garcìa-Diz L., Martìnez-Tomè M., Mariscal M., Murcia M.A. (2009). Influence of cooking methods on antioxidant activity of vegetables. J. Food Sci..

[35] Alizadeh-Pasdar N., Li-Chan E.C.Y. (2000). Comparison of protein surface hydrophobicity measured at various pH values using three different fluorescent probes. J. Agric. Food Chem..

[36] Singleton V.L., Rossi J.A. (1965). Colorimetry of total phenolics with phosphomolybdic-phosphotungstic acid reagents. Am. J. Enol. Vitic..

[37] Yen G.C., Chen H.Y. (1995). Antioxidant activity of various tea extracts in relation to their antimutagenicity. J. Agric. Food Chem..

[38] Kirby A.J., Schmidt R.J. (1997). The antioxidant activity of Chinese herbs for eczema and of placebo herbs. J. Ethnopharmacol..

